# Lung and bone metastases patterns in osteosarcoma: Chemotherapy improves overall survival

**DOI:** 10.1097/MD.0000000000032692

**Published:** 2023-01-27

**Authors:** Liyuan Tang, Binbin Liu

**Affiliations:** a Drug Clinical Trial Institution, Cangzhou Central Hospital, Cangzhou, Hebei, P.R. China; b Department of Orthopedics, Cangzhou Central Hospital, Cangzhou, Hebei, P.R. China.

**Keywords:** bone metastases, lung metastases, osteosarcoma, risk factors, SEER program, survival factors

## Abstract

Osteosarcoma (OS) is a malignant tumor originating from the mesenchymal tissue. Simultaneous reports of lung and bone metastases (BM) in OS are rare in the literature. A total of 353 new cases of lung metastases (LM), 93 new cases of BM, and 59 new cases of LM and BM were diagnosed in the Surveillance, Epidemiology and End Results (SEER) database from 2010 to 2019. Univariate and multivariate logistic regression analyses were used to identify risk factors for LM and/or BM, and Cox regression analyses were performed to identify the prognostic factors for LM and/or BM. Kaplan–Meier (K–M) curves and log-rank tests were used to analyze the overall survival of patients with LM and/or BM. LM was diagnosed in 353 patients. Female sex, tumor size >100 mm, telangiectatic OS type, central OS type, N1 stage, other locations, BM, surgical treatments, radiotherapy and chemotherapy were significantly correlated with LM. 93 patients were diagnosed with BM. 25 to 59 years old, T1 stage, presence of LM, liver metastases, radiotherapy, and surgical treatments were significantly correlated with the BM. 59 patients were diagnosed with LM and BM. The chondroblastic OS type, small cell OS type, T1 stage, N1 stage, other locations, liver metastases, radiotherapy, and surgical treatments were significantly correlated with LM and BM. Metastases, radiotherapy, and surgery at the primary site were significantly associated with LM and/or BM. Chemotherapy at the primary site has been shown to be effective in improving the survival rate of LM and/or BM. Of the OS patients with LM, 61.47% died, and older age, BM, no surgery, and no chemotherapy were harmful to survival. 72.04% of OS patients with BM died, and N1 stage, no surgery, and no chemotherapy were harmful for survival. 69.49% of OS patients with LM and BM died, and older age and no chemotherapy were harmful for survival.

## 1. Introduction

Osteosarcoma (OS) is a rare malignant bone tumor that originates from bone mesenchymal cells and can occur at any age, but it is more common in children and adolescents.^[1]^ The incidence is higher in men than in women, and the number of new cases of OS has increased at a rate of 0.4% per year over the past decade.^[2]^ Metastasis is the most common cause of treatment failure in OS. Lung metastasis (LM) and bone metastasis (BM) are the first and second most common metastatic types of OS, respectively. LM are the most common, accounting for approximately 80% of cases,^[3,4]^ bone metastases (BM) account for approximately 11% of all metastases,^[5,6]^ and all other metastases account for less than 10%. The 5-year overall survival rate for OS patients with LM is about 30%, while the 5-year overall survival rate for OS patients without metastases is about 70%.^[7,8]^ OS is one of the most harmful malignant bone cancers and has a poor prognosis.^[9]^

The Surveillance, Epidemiology, and End Results (SEER) database is a publicly accessible database that collects data on approximately 30% of the United States’ population.^[10]^ It provides risk data, and prognostic data were collected from 18 established cancer registries in the United States.^[11]^ The data from this study is publicly available in the national cancer institute’s Surveillance, Epidemiology and End Results (SEER) database at https://seer.cancer.gov/data/access.html. We analyzed information from the SEER database to investigate the prevalence and risk factors of OS with LM and/or BM. In addition, survival analysis was performed for OS with LM and/or BM to assess the prognostic factors. Before 2010, in the SEER database, there is currently no detailed information on the metastases of all malignant tumors. Our study started in 2010, and the latest data on OS in 2019 was published in the SEER database in 2022.^[12–14]^

## 2. Material and methods

### 2.1. Ethical statement

The study was approved by the Ethics Committee of Cangzhou Central Hospital.

The study is based on Helsinki Declaration and subsequent amendments.

### 2.2. Study population

Data were collected from the SEER database. Risk and prognostic data were collected from 18 cancer registries in the United States. SEER * Stat 8.4.0 software was used to collect the case listings.^[15]^ Before 2010, there was no detailed information on the metastases of all malignant tumors in the SEER database.^[16]^ We studied patients with OS between 2010 and 2019, The histological types of OS were limited to 9180/3, 9181/3, 9182/3, 9183/3, 9184/3, 9185/3, 9186/3, 9187/3, 9192/3, 9193/3, and 9194/3 according to the International Classification of Diseases for Oncology-3 (ICD-O-3); 9180/3, 9181/3, 9182/3, 9183/3, 9184/3, 9185/3, 9186/3, 9186/3, 9193/3, 9193/3, and 9194/3 represent OS NOS, chondroblastic OS, fibroblastic OS, telangiectatic OS, OS in Paget disease of bone, small cell OS, central OS, intraosseous well-differentiated OS, parosteal OS, periosteal OS and high-grade surface OS, respectively.^[17]^ The exclusion criteria were as follows: patients with OS from the SEER database outside of January 1, 2010, to December 31, 2019; OS was not the primary disease of the patients; and blank options for OS patients.^[18,19]^ From January 1, 2010, to December 31, 2019, a total of 1990 patients with OS were enrolled: 353 cases of LM, 93 cases of BM, and 59 cases of LM and BM were diagnosed with OS with metastasis.

### 2.3. Statistical analysis

The demographic and clinical characteristics of patients with OS were divided into: age (≤24, 25–59, and ≥60 years); sex (female and male); race (White, Black, American Indian/Alaska Native, Asian or Pacific Islander and unknown); tumor size (<50, 50–100, >100 mm and unknown); tumor differentiation grade (I, II, III, IV, and unknown); Location (extremities, axial and others); T stage (T0, T1, T2, T3, T4, and unknown); N stage (N0, N1 and unknown); M stage (M0, M1 and unknown); Histological types (OS [9180/3, 9181/3, 9182/3, 9183/3, 9184/3, 9185/3, 9186/3, 9187/3, 9192/3, 9193/3 and 9194/3]); absence or presence of lung, bone, brain, liver, LM and BM; surgery, radiation and chemotherapy. Differences in the prevalence of OS were analyzed using Pearson’s chi-squared test. The risk factors for OS were first determined using univariate logistic regression. Multivariate analysis was performed for the statistically significant risk factors (*P* < .05). The K–M curve and log-rank test were used to analyze the survival differences in patients with OS. Multivariate Cox proportional risk regression was performed according to the above factors, and *P* < .05 was considered significant.^[20]^ All statistical analyses were used the Social Science Statistical Software Package (SPSS) 25.0 (IBM, Armonk, NY), all survival charts were used GraphPad Prism 9 (GraphPad, Inc.). Statistical significance was defined as a 2-tailed *P* < .05.

## 3. Results

### 3.1. Prevalence of OS

A total of 1990 patients with OS were included in this study. Among these, there were 353 cases of OS with LM, and the incidence of LM was 17.74%. The prevalence rates of OS with LM at ≤ 24, 25 to 59, and ≥60 years of age were 18.43%, 14.26%, and 23.68%, respectively. The LM rate of patients aged ≥60 years was significantly higher than that of younger patients (χ^2^ = 9.710; *P* = .008), and the LM rate of female patients was significantly higher than that of male patients (χ^2^ = 10.026; *P* = .002), and there was no significant difference in the prevalence of OS with LM among different ethnic groups. There were 93 cases of OS with BM, with an incidence of BM was 4.67%. The BM rate of patients aged ≥60 years was significantly higher than that of younger patients (χ^2^ = 6.720; *P* = .035), and there was no significant difference in the prevalence of OS with BM between different sex and ethnic groups. There were 59 cases of OS with LM and BM, and the incidence of LM and BM was 2.96%. There was no significant difference in the prevalence of OS with LM and BM in different age, sex, and ethnic groups. There were 7 cases of OS with brain metastasis and 11 cases of OS with liver metastasis, with an incidence of 0.35% and 0.55%, respectively. The other pathological and clinical data are shown in Table 1.

**Table 1:** Baseline of the demographic and related clinical characteristics among osteosarcoma patients with and without lung and/or bone metastases (diagnosed between 2010 and 2019).

Subject characteristics	No. of osteosarcoma patients		*P* value	No. of osteosarcoma patients		*P* value	No. of osteosarcoma patients		*P* value
	With LM(n, %)	Without LM(n, %)		With BM(n, %)	Without BM(n, %)		With LM and BM(n, %)	Without LM and BM(n, %)	
**n**	353(17.74)	1637(82.26)		93(4.67)	1897(95.33)		59(2.96)	1931(97.04)	
**Age, in yrs**									
** ≤24**	227(18.43)	1005(81.57)	.008*	54(4.38)	1178(95.62)	.035*	38(3.08)	1194(96.92)	.639
** 25–59**	81(14.26)	487(85.74)		23(4.05)	545(95.95)		14(2.46)	554(97.54)	
** ≥60**	45(23.68)	145(76.32)		16(8.42)	174(91.58)		7(3.68)	183(96.32)	
**Sex**									
** Male**	130(14.71)	754(85.29)	.002*	38(4.30)	846(95.70)	.479	22(2.49)	862(97.51)	.263
** Female**	223(20.16)	883(79.84)		55(4.97)	1051(95.03)		37(3.35)	1069(96.65)	
**Race**									
** White**	263(17.72)	1221(82.28)	.603	71(4.78)	1413(95.22)	.951	46(3.10)	1438(96.90)	.821
** Black**	54(18.95)	231(81.05)		13(4.56)	272(95.44)		6(2.11)	279(97.89)	
** AI**	3(15.79)	16(84.21)		1(5.26)	18(94.74)		1(5.26)	18(94.74)	
** API**	33(17.28)	158(82.72)		8(4.19)	183(95.81)		6(3.14)	185(96.86)	
** Unknown**	0(0.00)	11(100.00)		0(0.00)	11(100.00)		0(0.00)	11(100.00)	
**Tumor size(mm**)									
** <50**	16(6.58)	227(93.42)	<.001*	5(2.06)	238(97.94)	.005*	3(1.23)	240(98.77)	.020*
** 50–100**	87(11.60)	663(88.40)		28(3.73)	722(96.27)		16(2.13)	734(97.87)	
** >100**	184(25.34)	542(74.66)		38(5.23)	688(94.77)		26(3.58)	700(96.42)	
** Unknown**	66(24.35)	205(75.65)		22(8.12)	249(91.88)		14(5.17)	257(94.83)	
**Histological type**									
** Osteosarcoma and NOS**	271(20.06)	1080(79.94)	<.001*	73(5.40)	1278(94.60)	.094	46(3.40)	1305(96.60)	.010*
** Chondroblastic osteosarcoma**	50(17.67)	233(82.33)		9(3.18)	274(96.82)		3(1.06)	280(98.94)	
** Fibroblastic osteosarcoma**	5(9.62)	47(90.38)		2(3.85)	50(96.15)		2(3.85)	50(96.15)	
** Telangiectatic osteosarcoma**	3(5.26)	54(94.74)		1(1.75)	56(98.25)		0(0.00)	57(100.00)	
** Osteosarcoma in Paget disease of bone**	2(18.18)	9(81.82)		1(9.09)	10(90.91)		1(9.09)	10(90.91)	
** Small cell osteosarcoma**	7(38.89)	11(61.11)		3(16.67)	15(83.33)		3(16.67)	15(83.33)	
** Central osteosarcoma**	7(8.05)	80(91.95)		1(1.15)	86(98.85)		1(1.15)	86(98.85)	
** Intraosseous well differentiated osteosarcoma**	0(0.00)	4(100.00)		0(0.00)	4(100.00)		0(0.00)	4(100.00)	
** Parosteal osteosarcoma**	4(4.12)	93(95.88)		2(2.06)	95(97.94)		2(2.06)	95(97.94)	
** Periosteal osteosarcoma**	2(10.00)	18(90.00)		0(0.00)	20(100.00)		0(0.00)	20(100.00)	
** High grade surface osteosarcoma**	2(20.00)	8(80.00)		1(10.00)	9(90.00)		1(10.00)	9(90.00)	
**T stage**									
** T0**	1(33.33)	2(66.67)	<.001*	2(66.67)	1(33.33)	<.001*	1(33.33)	2(66.67)	<.001*
** T1**	52(7.69)	624(92.31)		13(1.92)	663(98.08)		5(0.74)	671(99.26)	
** T2**	204(20.86)	774(79.14)		44(4.50)	934(95.50)		30(3.07)	948(96.93)	
** T3**	29(40.28)	43(59.72)		14(19.44)	58(80.56)		9(12.50)	63(87.50)	
** T4**	6(37.50)	10(62.50)		1(6.25)	15(93.75)		1(6.25)	15(93.75)	
** Unknown**	61(24.90)	184(75.10)		19(7.76)	226(92.24)		13(5.31)	232(94.69)	
**N stage**									
** N0**	299(16.22)	1544(83.78)	<.001*	77(4.18)	1766(95.82)	<.001*	46(2.50)	1797(97.50)	<.001*
** N1**	28(52.83)	25(47.17)		8(15.09)	45(84.91)		7(13.21)	46(86.79)	
** Unknown**	26(27.66)	68(72.34)		8(8.51)	86(91.49)		6(6.38)	88(93.62)	
**M stage**									
** M0**	0(0.00)	1558(100.00)	<.001*	0(0.00)	1558(100.00)	<.001*	0(0.00)	1558(100.00)	<.001*
** M1**	353(83.85)	68(16.15)		93(22.09)	328(77.91)		59(14.01)	362(85.99)	
** Unknown**	0(0.00)	11(100.00)		0(0.00)	11(100.00)		0(0.00)	11(100.00)	
**Grade**									
** Grade I**	1(1.12)	88(98.88)	<.001*	0(0.00)	89(100.00)	.016*	0(0.00)	89(100.00)	.077
** Grade II**	6(5.77)	98(94.23)		1(0.96)	103(99.04)		0(0.00)	104(100.00)	
** Grade III**	86(19.37)	358(80.63)		19(4.28)	425(95.72)		11(2.48)	433(97.52)	
** Grade IV**	122(18.51)	537(81.49)		29(4.40)	630(95.60)		21(3.19)	638(96.81)	
** Unknown**	138(19.88)	556(80.12)		44(6.34)	650(93.66)		27(3.89)	667(96.11)	
**Location**									
** Extremities**	270(17.66)	1259(82.34)	<.001*	55(3.60)	1474(96.40)	<.001*	36(2.35)	1493(97.65)	<.001*
** Axial**	73(27.76)	190(72.24)		29(11.03)	234(88.97)		21(7.98)	242(92.02)	
** Others**	10(5.05)	188(94.95)		9(4.55)	189(95.45)		2(1.01)	196(98.99)	
**Bone or/and Lung metastases**									
** None**	294(15.50)	1603(84.50)	<.001*	34(2.08)	1603(97.92)	<.001*	0(0.00)	1931(100.00)	NA
** Yes**	59(63.44)	34(36.56)		59(16.71)	294(83.29)		59(100.00)	0(0.00)	
** Unknown**	0(0.00)	0(0.00)		0(0.00)	0(0.00)		0(0.00)	0(0.00)	
**Brain metastases**									
** None**	348(17.56)	1634(82.44)	.002*	90(4.54)	1892(95.46)	<.001*	57(2.88)	1925(97.12)	<.001*
** Yes**	4(57.14)	3(42.86)		2(28.57)	5(71.43)		1(14.29)	6(85.71)	
** Unknown**	1(100.00)	0(00.00)		1(100.00)	0(00.00)		1(100.00)	0(00.00)	
**Liver metastases**									
** None**	347(17.53)	1632(82.47)	.001*	87(4.40)	1892(95.60)	<.001*	54(2.73)	1925(97.27)	<.001*
** Yes**	6(54.55)	5(45.45)		6(54.55)	5(45.45)		5(45.45)	6(54.55)	
** Unknown**	0(00.00)	0(00.00)		0(0.00)	0(00.00)		0(0.00)	0(00.00)	
**Surg**									
** None**	120(47.43)	133(52.57)	<.001*	47(18.58)	206(81.42)	<.001*	30(11.86)	223(88.14)	<.001*
** Yes**	232(13.46)	1492(86.54)		45(2.61)	1679(97.39)		29(1.68)	1695(98.32)	
** Unknown**	1(7.69)	12(92.31)		1(7.69)	12(92.31)		0(00.00)	13(100.00)	
**Radiation**									
** None/Unknown**	300(16.58)	1509(83.42)	<.001*	67(3.70)	1742(96.30)	<.001*	44(2.43)	1765(97.57)	<.001*
** Yes**	53(29.28)	128(70.72)		26(14.36)	155(85.64)		15(8.29)	166(91.71)	
**Chemotherapy**									
** No/Unknown**	31(9.57)	293(90.43)	<.001*	12(3.70)	312(96.30)	.366	5(1.54)	319(98.46)	.099
** Yes**	322(19.33)	1344(80.67)		81(4.86)	1585(95.14)		54(3.24)	1612(96.76)	
** Unknown**	2	1(50.00)	NA	NA	NA				

AI = American Indian/Alaska Native, API = asian or pacific islander, BM = bone metastases, LM = lung metastases, NA = not available, Surg = surgical treatments of primary site.

### 3.2. Risk factors for developing OS

Univariate logistic regression analysis revealed that multiple factors were significantly associated with LM. These items were: 25 to 59 years old (OR = 0.74, 95% CI: 0.56–0.97, *P* = .030), female (OR = 1.47, 95% CI: 1.16–1.86, *P* = .002); primary tumor size: 50 to 100 mm (OR = 1.86, 95% CI: 1.07–3.24, *P* = .028), >100 mm (OR = 4.82, 95% CI: 2.82–8.21, *P* < .001); telangiectatic OS (OR = 0.22, 95% CI: 0.07–0.71, *P* = .012); central OS (OR = 0.35, 95% CI:0.16-0.76, *P* = .008); parosteal OS (OR = 0.17, 95% CI: 0.06–0.47, *P* = .001); N1 stage (OR = 5.78, 95% CI: 3.33–10.06, *P* < .001); grade III (OR = 21.14, 95% CI: 2.90–153.89, *P* = .003); grade IV (OR = 19.99, 95% CI: 2.76–144.92, *P* = .003); BM (OR = 9.46, 95% CI: 6.09–14.69, *P* < .001); brain metastases (OR = 6.26, 95% CI: 1.40–28.10, *P* = .017); liver metastases (OR = 5.64, 95% CI: 1.71–18.60, *P* = .004); location: axial (OR = 1.79, 95% CI: 1.33–2.42, *P* < .001), others (OR = 0.25, 95% CI: 0.13–0.48, *P* < .001); surgical treatments of primary site (OR = 0.17, 95% CI: 0.13–0.23, *P* < .001); radiation (OR = 2.08, 95% CI: 1.48–2.94, *P* < .001) and chemotherapy (OR = 2.26, 95% CI: 1.53–3.34, *P* < .001) were significantly correlated with LM. Multivariate logistic regression analysis showed that multiple factors were significantly associated with LM. These items were: female (OR = 1.40, 95% CI: 1.07–1.83, *P* = .015); primary tumor size >100 mm (OR = 2.80, 95% CI: 1.56–5.03, *P* = .001); telangiectatic OS (OR = 0.23, 95% CI: 0.07–0.80, *P* = .020); central OS (OR = 0.25, 95% CI: 0.18–0.89, *P* = .025); N1 stage (OR = 4.65, 95% CI: 2.40–9.01, *P* < .001); other location (OR = 0.19, 95% CI: 0.08–0.42, *P* < .001); BM (OR = 4.89, 95% CI: 2.93–8.16, *P* < .001); surgical treatments of primary site (OR = 0.20, 95% CI: 0.14–0.29, *P* < .001); radiation (OR = 1.89, 95% CI: 1.18–3.03, *P* = .008) and chemotherapy (OR = 2.15, 95% CI: 1.24–3.71, *P* = .006) were significantly correlated with LM. The risk factors for LM are shown in Table 2.

**Table 2:** Multivariable logistic regression analysis of characteristics of osteosarcoma patients with lung and/or bone metastases (diagnosed between 2010 and 2019).

Subject characteristics	Osteosarcoma patients with lung metastases		Osteosarcoma patients with bone metastases			Osteosarcoma patients with lung and bone metastases	
	OR (95% CI)	*P* value	OR (95% CI)	*P* value		OR (95% CI)	*P* value
**Age, in yrs**							
** ≤24**	1(Reference)	1.00	1(Reference)	1.00		NA	NA
** 25–59**	0.77(0.54–1.08)	.130	0.50(0.27–0.94)	.031*		NA	NA
** ≥60**	1.11(0.65–1.90)	.703	0.63(0.30–1.33)	.224		NA	NA
**Sex**							
** Male**	1(Reference)	1.00	NA	NA		NA	NA
** Female**	1.40(1.07–1.83)	.015*	NA	NA		NA	NA
**Tumor size (mm**)							
** <50**	1(Reference)	1.00	1(Reference)	1.00		1(Reference)	1.00
** 50–100**	1.19(0.65–2.18)	.573	2.30(0.65–8.16)	.198		1.03(0.21–5.13)	.972
** >100**	2.80(1.56–5.03)	.001*	1.55(0.40–5.98)	.527		0.86(0.16–4.57)	.855
** Unknown**	NA	NA	NA	NA		NA	NA
**Histological type**							
** Osteosarcoma and NOS**	1(Reference)	1.00	NA	NA		1(Reference)	1.00
** Chondroblastic osteosarcoma**	0.89(0.61–1.29)	.536	NA	NA		0.28(0.08–0.96)	.043*
** Fibroblastic osteosarcoma**	0.60(0.21–1.72)	.344	NA	NA		2.42(0.53–11.13)	.255
** Telangiectatic osteosarcoma**	0.23(0.07–0.80)	.020*	NA	NA		0.00(0.00–NA)	.997
** Osteosarcoma in Paget disease of bone**	0.28(0.04–1.95)	.199	NA	NA		0.59(0.04–8.12)	.692
** Small cell osteosarcoma**	2.12(0.72–6.29)	.175	NA	NA		5.60(1.35–23.16)	.017*
** Central osteosarcoma**	0.40(0.18–0.89)	.025*	NA	NA		0.49(0.06–3.75)	.489
** Intraosseous well differentiated osteosarcoma**	0.00(0.00–NA)	.999	NA	NA		0.00(0.00–NA)	.999
** Parosteal osteosarcoma**	0.65(0.20–2.06)	.463	NA	NA		1.30(0.29–5.81)	.728
** Periosteal osteosarcoma**	1.30(0.28–6.10)	.742	NA	NA		0.00(0.00–NA)	.998
** High grade surface osteosarcoma**	0.78(0.12–5.21)	.797	NA	NA		2.37(0.23–24.87)	.472
**T stage**							
** T0**	NA	NA	1(Reference)	1.00		1(Reference)	1.00
** T1**	NA	NA	0.03(0.00–0.62)	.023*		0.02(0.00–0.72)	.033*
** T2**	NA	NA	0.07(0.00–1.34)	.077		0.07(0.00–3.15)	.169
** T3**	NA	NA	0.22(0.01–4.27)	.314		0.22(0.01–10.26)	.437
** T4**	NA	NA	0.03(0.00–1.24)	.065		0.04(0.00–3.87)	.171
** Unknown**	NA	NA	NA	NA		NA	NA
**N stage**							
** N0**	1(Reference)	1.00	1(Reference)	1.00		1(Reference)	1.00
** N1**	4.65(2.40–9.01)	<.001*	1.62(0.64–4.06)	.309		3.98(1.48–10.74)	.006*
** Unknown**	NA	NA	NA	NA		NA	NA
**Grade**							
** Grade I**	1(Reference)	1.00	NA	NA		NA	NA
** Grade II**	2.95(0.32–27.22)	.339	NA	NA		NA	NA
** Grade III**	5.44(0.67–44.20)	.113	NA	NA		NA	NA
** Grade IV**	4.53(0.56–36.74)	.158	NA	NA		NA	NA
** Unknown**	NA	NA	NA	NA		NA	NA
**Location**							
** Extremities**	1(Reference)	1.00	1(Reference)	1.00		1(Reference)	1.00
** Axial**	0.80(0.52–1.21)	.286	1.55(0.82–2.94)	.179		1.48(0.73–3.01)	.276
** Others**	0.19(0.08–0.42)	<.001*	1.52(0.55–4.18)	.417		0.09(0.01–0.89)	.039*
**Bone or/and Lung metastases**							
** None**	1(Reference)	1.00	1(Reference)	1.00		NA	NA
** Yes**	4.89(2.93–8.16)	<.001*	5.20(3.10–8.71)	<.001*		NA	NA
** Unknown**	NA	NA	NA	NA		NA	NA
**Brain metastases**							
** None**	1(Reference)	1.00	1(Reference)	1.00		NA	NA
** Yes**	3.17(0.45–22.41)	.248	3.62(0.59–22.35)	.165		NA	NA
** Unknown**	NA	NA	NA	NA		NA	NA
**Liver metastases**							
** None**	1(Reference)	1.00	1(Reference)	1.00		1(Reference)	1.00
** Yes**	1.85(0.38–8.99)	.446	8.08(1.81–36.17)	.006*		18.65(3.70–94.03)	<.001*
** Unknown**	NA	NA	NA	NA		NA	NA
**Surg**							
** None**	1(Reference)	1.00	1(Reference)	1.00		1(Reference)	1.00
** Yes**	0.20(0.14–0.29)	<.001*	0.29(0.17–0.52)	<.001*		0.23(0.12–0.43)	<.001*
** Unknown**	NA	NA	NA	NA		NA	NA
**Radiation**							
** None/Unknown**	1(Reference)	1.00	1(Reference)	1.00		1(Reference)	1.00
** Yes**	1.89(1.18–3.03)	.008*	3.02(1.67–5.49)	<.001*		2.64(1.27–5.49)	.010*
**Chemotherapy**							
** No/Unknown**	1(Reference)	1.00	NA	NA		NA	NA
** Yes**	2.15(1.24–3.71)	.006*	NA	NA		NA	NA
** Unknown**	2	1(50.00)	NA		NA		NA

AI = American Indian/Alaska Native, API = asian or pacific islander, NA = not available, Surg = surgical treatments of primary site.

* statistically significant.

Univariate logistic regression analysis revealed that multiple factors were significantly associated with BM. These items were ≥ 60 years old (OR = 2.01, 95% CI: 1.12–3.58, *P* = .019), primary tumor size: >100 mm (OR = 2.63, 95% CI: 1.02–6.76, *P* = .045); T1 stage (OR = 0.01 95% CI: 0.00–0.12, *P* < .001); T2 stage (OR = 0.02, 95% CI:0.00–0.27, *P* = .002); T4 stage (OR = 0.03 95% CI: 0.00–0.77, *P* = .034); N1 stage (OR = 4.08, 95% CI: 1.86–8.95, *P* < .001); brain metastases (OR = 8.41, 95% CI: 1.61–43.93, *P* = .012); liver metastases (OR = 26.10, 95% CI: 7.81–87.18, *P* < .001); LM (OR = 9.46, 95% CI: 6.09–14.69, *P* < .001); location: axial (OR = 3.32, 95% CI: 2.08–5.32, *P* < .001); surgical treatments of primary site (OR = 0.12, 95% CI: 0.08–0.18, *P* < .001); radiation (OR = 4.36, 95% CI: 2.69–7.06, *P* < .001) were significantly correlated with BM. Multivariate logistic regression analysis showed that multiple factors were significantly associated with BM. These items were as follows: 25 to 59 years old (OR = 0.50, 95% CI: 0.27–0.94, *P* = .031); T1 stage (OR = 0.03 95% CI: 0.00–0.62, *P* = .023); liver metastases (OR = 8.08, 95% CI: 1.81–36.17, *P* = .006); LM (OR = 5.20, 95% CI: 3.10–8.71, *P* < .001); surgical treatments of the primary site (OR = 0.29, 95% CI: 0.17–0.52, *P* < .001); and radiation (OR = 3.02, 95% CI: 1.67–5.49, *P* < .001) were significantly correlated with BM. The risk factors for the BM are shown in Table 2.

Univariate logistic regression analysis showed that multiple factors were significantly associated with LM and BM. These items were: primary tumor size, chondroblastic OS (OR = 0.30, 95% CI: 0.09–0.98, *P* = .047); small cell OS (OR = 5.67 95% CI: 1.59–20.29, *P* = .008); T1 stage (OR = 0.02 95% CI: 0.00–0.19, *P* = .001); T2 stage (OR = 0.06 95% CI: 0.00–0.72, *P* = .026); N1 stage (OR = 5.95, 95% CI: 2.55–13.87, *P* < .001); axial location (OR = 3.60, 95% CI: 2.07–6.27, *P* < .001); liver metastases (OR = 29.71, 95% CI: 8.80–100.35, *P* < .001); surgical treatments of primary site (OR = 0.13, 95% CI: 0.08–0.22, *P* < .001); radiation (OR = 3.63, 95% CI: 1.98–6.65, *P* < .001); and chemotherapy (OR = 2.26, 95% CI: 1.53–3.34, *P* < .001) were significantly correlated with the LM and BM. Multivariate logistic analysis showed that multiple factors were significantly associated with LM and BM. These items were chondroblastic OS (OR = 0.28, 95% CI: 0.08–0.96, *P* = .043); small cell OS (OR = 5.60, 95% CI: 1.35–23.16, *P* = .017); T1 stage (OR = 0.02 95% CI: 0.00–0.72, *P* = .033); N1 stage (OR = 3.98 95% CI: 1.48–10.74, *P* = .006); other locations (OR = 0.09, 95% CI: 0.01–0.89, *P* = .039); liver metastases (OR = 18.65, 95% CI: 3.70–94.03, *P* < .001); surgical treatments of the primary site (OR = 0.23, 95% CI: 0.12–0.43, *P* < .001); and radiation (OR = 2.64, 95% CI: 1.27–5.49, *P* = .010) were significantly correlated with LM and BM. The risk factors for the BM are shown in Table 2.

### 3.3. Survival time and prognostic factors for OS

**Figure 1.**
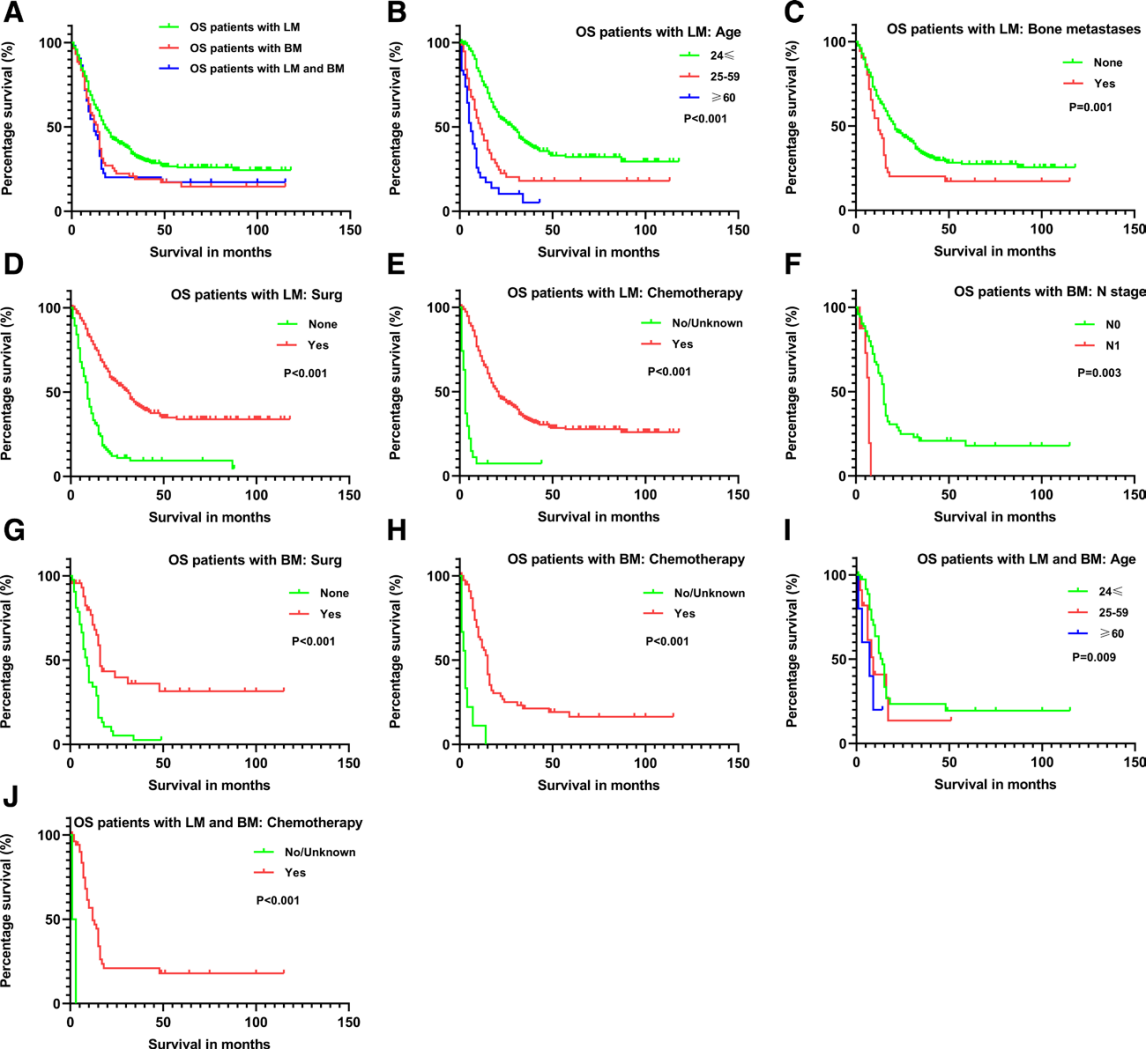
Kaplan–Meier analysis was performed on osteosarcoma patients with lung and/or bone metastases: (A) overall, osteosarcoma patients with lung metastases: (B) age, (C) bone metastasis, (D) surgical treatments of the primary site and (E) chemotherapy, osteosarcoma patients with bone metastases: (F) N stage, (G) surgical treatments of the primary site and (H) chemotherapy, osteosarcoma patients with lung and bone metastases: (I) age and (J) chemotherapy. BM = bone metastases, LM = lung metastases, OS = osteosarcoma, Surg = surgical treatments of the primary site.

Table 3 shows prognostic factors of OS. Overall survival time of the K–M analysis was shown in Figure 1A. 61.47% (N = 217) of OS patients with LM died. K–M analysis of overall survival showed that OS patients with LM aged 25 to 59 years old, ≥60 years old (Fig. 1B, *P* < .001), histological type (chondroblastic OS, fibroblastic OS, telangiectatic OS, OS in Paget disease of bone, small cell OS, central OS, parosteal OS, periosteal OS and high-grade surface OS, *P* = .017), location (axial and others, *P* < .001), N1 stage (*P* = .003), with BM (Fig. 1C, *P* = .001), brain metastases (*P* = .024), liver metastases (*P* = .021) and radiation (*P* = .001) were lower than their parallel projects, and the patients who underwent surgical treatment at the primary site (Fig. 1D, *P* < .001) and chemotherapy (Fig. 1E, *P* < .001) were higher than their parallel projects (*P* < .001). In the multivariable Cox regression model, the overall survival rate of elderly patients (25–59 years old, hazard ratio [HR] = 1.72, 95% CI: 1.16–2.54, *P* = .007; ≥60 years old, HR = 3.46, 95% CI: 2.16–5.54, *P* < .001), BM (HR = 2.10, 95% CI: 1.40–3.16, *P* < .001) were harmful for survival, with mean survival times of 11, 5, and 12 months, respectively.

**Table 3:** Multivariate Cox regression analysis of prognostic factors in osteosarcoma patients with lung and/or bone metastases (diagnosed between 2010 and 2019).

Subject characteristics	No. of osteosarcoma patients with lung or/and bone metastases		Mean survival mouth	HR (95% CI)	*P* value
	Overall	Deceased (n, %)			
**Osteosarcoma patients with lung metastases**	353	217(61.47)			
**Age, in yrs**					
** ≤24**	227	124(54.63)	28.00(21.71–34.29)	1(Reference)	1.00
** 25–59**	81	54(66.67)	11.00(7.70–14.30)	1.72(1.16–2.54)	.007
** ≥60**	45	39(86.67)	5.00(3.15–6.85)	3.46(2.16–5.54)	<.001
**Bone metastases**					
** None**	294	176(59.86)	21.00(16.90–25.11)	1(Reference)	1.00
** Yes**	59	41(69.49)	12.00(8.20–15.80)	2.10(1.40–3.16)	<.001
** Unknown**	0	0(00.00)	NA	NA	NA
**Surg**					
** None**	120	99(82.50)	9.00(7.57–10.43)	1(Reference)	1.00
** Yes**	232	118(50.86)	31.00(25.07–36.93)	0.44(0.31–0.63)	<.001
** Unknown**	1	0(0.00)	NA	NA	NA
**Chemotherapy**					
** No/Unknown**	31	28(90.32)	3.00(2.28–3.72)	1(Reference)	1.00
** Yes**	322	189(58.70)	20.00(16.33–23.68)	0.22(0.13–0.37)	<.001
** Osteosarcoma patients with bone metastases**	93	67(72.04)			
**N stage**					
** N0**	77	54(70.13)	15.00(13.52–16.48)	1(Reference)	1.00
** N1**	8	6(75.00)	7.00(6.14–7.86)	4.29(1.40–13.13)	.011
** Unknown**	8	7(87.50)	NA	NA	NA
**Surg**					
** None**	45	43(95.56)	8.00(5.57–10.43)	1(Reference)	1.00
** Yes**	47	23(48.94)	16.00(13.83–18.17)	0.36(0.18–0.71)	.003
** Unknown**	1	1(100.00)	NA	NA	NA
**Chemotherapy**					
** No/Unknown**	12	11(91.67)	2.00(0.00–4.12)	1(Reference)	1.00
** Yes**	81	56(69.14)	15.00(12.74–17.26)	0.18(0.07–0.47)	<.001
** Osteosarcoma patients with lung and bone metastases**	59	41(69.49)			
**Age, in years**					
** ≤24**	38	25(65.79)	14.00(11.09–16.91)	1(Reference)	1.00
** 25–59**	14	10(71.43)	8.00(2.66–13.35)	1.48(0.18–12.34)	.717
** ≥60**	7	6(85.71)	3.00(0.00–8.13)	8.02(1.14–56.34)	.036
**Chemotherapy**					
** No/Unknown**	5	4(80.00)	1.00(0.00–2.56)	1(Reference)	1.00
** Yes**	54	37(68.52)	12.00(7.62–16.39)	0.00(0.00–0.15)	.008

NA = not available, Surg = surgical treatments of the primary site.

The results showed that patients who underwent primary site surgery (HR = 0.44, 95% CI: 0.31–0.63, *P* < .001) and chemotherapy (HR = 0.22, 95% CI: 0.13–0.37, *P* < .001) had better overall survival than those who did not undergo surgical treatment and chemotherapy, with mean survival times of 31 and 20 months, respectively.

A total of 72.04% (N = 67) of patients with BM died. K-M analysis of overall survival showed that the patients aged 25 to 59 years old and ≥60 years old (*P* < .001), primary tumor size: 50 to 100 mm and >100 mm (*P* < .001), location (axial and others, *P* < .001), N1 stage (Fig. 1F, *P* = .003), higher T stage (*P* < .001), and radiation (*P* = .028) were lower than those of parallel projects, and the patients who underwent surgical treatment at the primary site (Fig. 1G, *P* < .001) and chemotherapy (Fig. 1H, *P* < .001) were higher than those of parallel projects. In the multivariable Cox regression model, the overall survival rate of N1 stage (HR = 4.29, 95% CI: 1.40–13.13, *P* = .011) was harmful for survival with a mean survival time of 7 months. The results showed that patients undergoing primary site surgery (HR = 0.36, 95% CI: 0.18–0.71, *P* = .003) and chemotherapy (HR = 0.18, 95% CI: 0.07–0.47, *P* < .001) had better overall survival than those who did not undergo surgical treatment and chemotherapy, with mean survival times of 16 and 15 months, respectively. The prognostic factors for BM are shown in Table 3.

A total of 69.49% (N = 41) of patients with LM and BM died. K-M analysis of overall survival showed that patients aged 25 to 59 years old and ≥60 years old (Fig. 1I, *P* = .009), primary tumor size: 50 to 100 mm and >100 mm (*P* = .020), location (axial and others, *P* < .001), histological type (chondroblastic OS, fibroblastic OS, OS in Paget disease of bone, small cell OS, central OS, parosteal OS, and high-grade surface OS, *P* = .044), N1 stage (*P* = .049), and higher T stage (*P* < .001) were lower than those of parallel projects, and the patients who underwent surgical treatment at the primary site (*P* < .001) and chemotherapy (Fig. 1J, *P* < .001) were higher than those in parallel projects. In the multivariable Cox regression model, the overall survival rate of elderly patients (≥60 years old, HR = 8.02, 95% CI: 1.14–56.34, *P* = .036) was harmful for survival, with a mean survival time of 3 months. The results showed that patients undergoing chemotherapy (HR = 0.00, 95% CI: 0.00–0.15, *P* = .008) had better overall survival than those who did not undergo surgical treatment and chemotherapy, with a mean survival time of 12 months. The prognostic factors for the BM are shown in Table 3.

## 4. Discussion

This study aimed to evaluate the risk and prognosis factors associated with OS patients with LM and/or BM. There are many reports of OS that has spread to the lung and several reports of OS that has spread to the bone; however, studies reporting both LM and BM are uncommon. Metastasis is the leading cause of treatment failure in OS. Approximately 80% of OS patients had LM during the course of treatment, and 14% to 20% of these patients had other metastases at the same time.^[21]^ We found 4.67% of patients with OS had BM, 1.61% had LM only, LM and BM accounted for 2.96% of all patients with OS, with approximately 0.30% of these patients having both liver or brain metastases. Patients with combined LM and BM only accounted for 2.66% of all OS patients.^[22]^ We found that LM was a risk factor for OS combined with BM, and BM was a risk factor for OS combined with LM, ang liver metastasis was a risk factor for OS combined with BM and OS combined with LM and BM. Radiotherapy and surgery are recognized protective factors and are considered an effective basic treatments for primary and recurrent metastatic OS. We found that both surgery and radiotherapy were risk factors for OS with LM and/or BM.^[23]^ Surgery is a recognized protective factor and has been recognized as an effective primary treatment for metastatic OS.^[24]^ This study found that surgery improved the prognostic factors of OS combined with LM or BM; however, OS combined with LM and BM was not. OS is often treated with chemotherapy followed by surgery; therefore, the tumor’s response to chemotherapy should be evaluated before surgical resection is determined to reduce the risk of recurrence.^[25,26]^ This study found chemotherapy to be an effective treatment for OS of the lung and/or bone in general.^[27]^

This study had several limitations. First, there is no more information about OS in the SEER database, including details such as the location of OS (cervical, thoracic, lumbar vertebrae, femur, and tibial bone), and presence of neurological symptoms.^[28]^ Second, in patients with OS, the SEER database did not record asymptomatic patients or those who developed advanced metastases. Third, the SEER database did not provide further surgical information, including details such as the type of surgery, time of surgery, and intraoperative blood loss. Finally, this study did not take into account marital history, income, and insurance coverage, which may have influenced survival among patients with OS.^[29]^

## 5. Conclusion

Metastases, radiotherapy and surgery at the primary site were significantly associated with LM and/or BM. Chemotherapy at the primary site has been shown to be effective in improving the survival rate of LM and/or BM.

## Acknowledgments

The authors are grateful to all participating patients.

## Author contributions

**Conceptualization:** Liyuan Tang, Binbin Liu.

**Data curation:** Liyuan Tang, Binbin Liu.

**Formal analysis:** Liyuan Tang, Binbin Liu.

**Investigation:** Liyuan Tang.

**Methodology:** Liyuan Tang.

**Project administration:** Liyuan Tang.

**Software:** Liyuan Tang.

**Supervision:** Liyuan Tang.

**Validation:** Liyuan Tang.

**Visualization:** Liyuan Tang.

**Writing – original draft:** Liyuan Tang.

**Writing – review & editing:** Liyuan Tang.
